# Impulse Dyscontrol Symptoms Are Related to Amyloid Beta in Patients With Subjective Cognitive Decline and Mild Cognitive Impairment

**DOI:** 10.1111/psyg.70119

**Published:** 2025-12-02

**Authors:** Ana Luiza Gonçalves Rochetti, Isadora Cristina Ribeiro, Lucas Luchesi Barnes, Liara Rizzi, Italo Karmann Aventurato, Brenda Costa Gonçalves, Marjorie Cristina Rocha da Silva, Marcio Luiz Figueredo Balthazar

**Affiliations:** ^1^ School of Medical Sciences, UNICAMP/SP Campinas Brazil

## Abstract

**Introduction:**

The relationship between neuropsychiatric symptoms (NPSs) and Alzheimer's disease (AD) pathophysiology, considering cerebrospinal fluid (CSF) biomarkers, is still not clarified and the available results in the literature are contradictory especially when it comes to the prodromic stages of AD. Clarifying the role of the AD biomarkers in the development of NPS in subjective cognitive decline (SCD) and mild cognitive impairment (MCI) is relevant and can be useful for planning longitudinal interventions in the AD continuum. The present work studies the correlation between NPSs and AD CSF biomarkers in SCD and MCI Brazilian subjects.

**Methods:**

This is a transversal study designed to evaluate the relationship between NPS and CSF AD biomarkers in SCD and MCI individuals. Voluntary participants were recruited through a virtually announced formulary. The inclusion criteria were age 55 years or older, of both sexes, without distinction of ethnicity or socioeconomic status, the presence of cognitive complaints, and that were willing to participate in all research procedures. Exclusion criteria for all subjects included clinical dementia, other neurological or psychiatric diseases, traumatic brain injury that resulted in a loss of consciousness, drug or alcohol addiction, prior chronic exposure to neurotoxic substances, Fazekas scale ≥ 2, and a score on the Montreal Cognitive Assessment (MoCA) less than 17 points. All participants had Pfeffer's Functional Activities Questionnaire < 5 and underwent medical and neuropsychological evaluation.

**Results:**

Seventy‐one patients were included between 2019 and 2022, 54 with MCI and 17 with SCD. A significant correlation (*p*: 0.006) was observed between MBI‐C C domain and the concentrations of CSF amyloid‐beta peptide.

**Conclusion:**

The study identified a significant correlation between domain C of the MBI‐C and CSF beta‐amyloid peptide analysing Brazilian patients with SCD and MCI. The results reinforce the hypothesis of the relationship between impulsivity symptoms and amyloid pathology. Further studies involving the correlations between MBI and neuropsychiatric symptoms with AD CSF biomarkers are necessary.

## 1 Introduction

Alzheimer's disease (AD) is biologically defined by the presence of neuritic plaques of amyloid‐beta peptide (A42) in the cerebral cortex and by neurofibrillary tangles of hyperphosphorylated tau (P‐tau) protein, which may be measured in vivo through Positron Emission Tomography Scan (PET‐Scan). The deposition of these proteins in the brain may lead to progressive cognitive symptoms, such as memory deficits in typical clinical presentation of AD and may cause loss of independence in daily activities and ultimately dementia [1].

The recent understanding of clinical aspects of AD proposes a *continuum* of cognitive symptoms initially with a pre‐clinical stage called subjective cognitive decline (SCD), then a prodromic stage called mild cognitive impairment (MCI), both being considered pre‐dementia stages. SCD is characterised by normal cognitive performance, with a subjective self‐perception of cognitive decline. MCI consists of impaired cognitive performance for age and education, with preservation of independence in daily assignments [1].

Cognitive staging is relevant to AD early diagnosis and treatment. Since many AD disease‐modifying therapy trials failed to demonstrate slowing down of the disease when given in dementia stages [2], it has become clear that the best moment to intervene in the amyloid cascade is in predementia stages [3]. Correctly identifying people at risk to develop dementia has become crucial to better understanding the course of symptoms and better selecting individuals for clinical trials.

Beyond cognitive presentations, neuropsychiatric symptoms (NPSs) are also significant in neurodegenerative diseases, and include abnormalities of behaviour, thought, mood, personality and others. In the AD continuum, NPS are significantly prevalent and associated with higher levels of morbidity, functional impairments, early and longer institutionalisation and caregiver overload [4].

Up to 97% of patients with AD dementia will present at least one NPS during the disease course [5]. NPS are also prevalent in non‐dementia individuals. Gallagher et al. [9] demonstrated that 30%–40% of the MCI subjects had apathy and 40%–50% of them presented depression and anxiety symptoms [6].

In this context, the concept of mild behavioural impairment (MBI) was developed to assess with higher accuracy NPS in predementia stages. MBI is a group of late‐onset neuropsychiatric and behavioural symptoms (≥ 50 years of age), with a consistency of ≥ 6 months, that cannot be classified according to a specific psychiatric syndrome [7]. Studies have shown that MBI can precede cognitive impairment in neurodegenerative diseases and is also associated with an increased risk of cognitive decline and progression to dementia [8]. MBI can be assessed with a specific scale, the mild behavioural impairment checklist (MBI‐C). MBI‐C is a questionnaire used in the diagnosis of MBI and has proven useful in the diagnosis of this syndrome in SCD and MCI patients. Each of its five domains (A–E) aims to map, respectively, a set of neuropsychiatric and behavioural symptoms: decreased motivation, emotional dysregulation, impulse dyscontrol, social inappropriateness and abnormalities in perception or thought content [9].

MBI‐C has proven to be more appropriate to evaluate NPS in SCD and MCI individuals, once it was elaborated specifically for this population, according to ISTAART‐AA criteria to MBI, with the objective to identify the symptoms with higher potential to be associated with cognitive decline [8]. Thus, the most common scales that measure NPS, such as the Neuropsychiatric Inventory (NPI) and its shorter version (NPI‐Questionnaire), may not be appropriate for predementia stages [10, 11]. These scales were developed to measure NPS in dementia and throughout only the month before evaluation. The questions are directed to the informant and may not capture nuances in behaviour, which could lead to less specificity, and may overestimate NPS prevalence due to its transient nature [12, 13].

The relationship between NPS and AD pathophysiology, considering CSF biomarkers, is still not clarified and the available results in the literature are contradictory especially when it comes to the prodromic stages of AD. A study with cognitively normal individuals at higher risk for developing AD, showed that lower levels of A42 and tau were associated with less NPS and neuroticism [14]. However, NG et al. (2021) study attested to a more significant role of A42 peptide in the manifestation of NPS in preclinical AD stages, when compared to tau protein [15].

Therefore, clarifying the role of the AD biomarkers in the development of NPS in SCD and MCI is relevant and can be useful for planning longitudinal interventions in the AD continuum. The present work studies the correlation between NPSs and AD cerebrospinal fluid (CSF) biomarkers in SCD and MCI Brazilian subjects.

## 2 Methods

### 2.1 Design

This is a transversal study designed to evaluate the relationship between NPSs and CSF AD biomarkers in SCD and MCI individuals. Voluntary participants were recruited through a virtually announced formulary. The inclusion criteria were age 55 years or older, of both sexes, without distinction of ethnicity or socioeconomic status, presence of cognitive complaints, and that they were willing to participate in all research procedures. Exclusion criteria for all subjects included clinical dementia, other neurological or psychiatric diseases, traumatic brain injury that resulted in a loss of consciousness, drug or alcohol addiction, prior chronic exposure to neurotoxic substances, Fazekas scale ≥ 2 and a score on the Montreal Cognitive Assessment (MoCA) less than 17 points [11]. Pre‐diagnostic procedures also comprised laboratory tests, including vitamin B12, folate, nontreponemal test for syphilis and thyroid hormones. All participants had Pfeffer's Functional Activities Questionnaire < 5 and underwent medical and neuropsychological evaluation.

### 2.2 SCD and MCI Criteria

SCD subjects were diagnosed using the core criteria of the SCD‐I Working Group Criteria for SCD [16], modified. We included individuals with memory complaints or persistent concerns of SCD for at least 1 year or who reported having a memory complaint and that this complaint interferes with their daily activities on the Memory Complaint Scale (MCS) [17]. All SCD individuals had cognitive performance within the average for age and education in all neuropsychological tests.

Regarding MCI, individuals were diagnosed using the core criteria of the NIA/AA for MCI [1]. We considered altered neuropsychological performance when they obtained a *z*‐score < −1.5 in any neuropsychological test; or obtained a *z*‐score between −1 and −1.5 in at least two neuropsychological tests that measured the same cognitive function; or received a *z*‐value score between −1 and −1.5 in at least three neuropsychological tests that measured different cognitive functions. All tests are validated for the Brazilian population, and age and education were considered to classify performance [14].

### 2.3 Cognitive Assessments

The neuropsychological tests used to confirm cognitive decline included the MoCA, the Clock Drawing Test (CDT), Rey's Auditory‐Verbal Learning Test (RAVLT); phonemic verbal fluency tests (words beginning with ‘F’, ‘A’ and ‘S’) and semantic verbal fluency tests (animal names); the Rey‐Osterrieth Complex Figure (ROCF); Trail Making Test A (TMT‐A) and B (TMT‐B), which refer to executive functions and cognitive processing speed; and the Boston Naming Test (BNT).

### 2.4 Neuropsychiatric Assessment

The following neuropsychiatric questionnaires were applied: Beck Depression Inventory (BDI‐II), Beck Anxiety Inventory (BAI) and MBI‐C. The BDI‐II consists of 21 sets of statements about depressive symptoms in the last 15 days that are rated on a 0‐to‐3 ordinal scale, yielding total scores ranging from 0 to 63. The suggested threshold for levels of severity was as follows: 0–13, minimal/no depression; 14–19, mild depression; 20–28, moderate depression; and 29–63, severe depression [18, 19]. The BAI comprises anxiety symptoms within the last week with 21 self‐rating items scored as in a Likert scale from 0 to 3. Scores may range from 0 to 63: 0–7, minimal anxiety levels; 8–15, mild anxiety; 16–25, moderate anxiety; and 26–63, severe anxiety [20, 21, 22].

The MBI‐C is a 34‐item instrument that can be completed by a clinician, the patient, or a close informant. It evaluates 5 domains: (A) decreased motivation (e.g., apathy, aspontaneity and indifference), with 6 questions; (B) emotional dysregulation (e.g., anxiety, dysphoria, changeability, euphoria and irritability), with 6 questions; (C) impulse dyscontrol (e.g., agitation, disinhibition, gambling, obsessiveness, behavioural perseveration and stimulus bind), with 12 questions; (D) social inappropriateness (e.g., lack of empathy, loss of insight, loss of social graces or tact, rigidity and exaggeration of previous personality traits), with 5 questions; and (E) abnormal perception or thought content (e.g., delusions and hallucinations) with 5 questions. Each question has a ‘Yes’ or ‘No’ answer, and then a severity score from 1 to 3 is given if there is a change regarding that question [9]. The final scores range from 0 to 102.

### 2.5 CSF AD Biomarkers Analysis

Samples of 4 mL of CSF collected from participants (SCD and MCI group) were used. The sample was centrifuged at 700 rpm and then stored at −80°C until the analyses were performed. The quantification of T‐tau, P‐tau_181_ and Aβ1‐42 proteins was performed by immunoassays in the Roche Cobas analyser. The following diagnostic kits were used: Elecsys total‐tau (T‐tau) CSF, Elecsys phospho‐tau (181P) CSF and Elecsys β‐Amyloid (1–42) CSF II. The reference values used to classify AT(N) were: βA < 1000 pg/mL, P‐tau > 27 pg/mL; T‐tau > 300 pg/mL.

### 2.6 Statistical Analysis

SPSS v25 program was used for the statistical analysis. Kolmogorov–Smirnov test was used to verify the distribution pattern of the sample. Student's *t*‐test was used to attest the variance of normal variables, and Mann Whitney *U* test was used to attest the variance of non‐parametric variables between SCD and MCI groups. Spearman correlation was used to verify the association between the neuropsychiatric scores and the biomarkers, adjusted for sex and age. A *p*‐value < 0.05 was considered statistically significant.

### 2.7 Ethics Considerations and Consent Statement

All subjects signed the informed consent form, and the study was approved by the University of Campinas Research Ethics Committee (CAAE No. 24898619.8.0000.5404).

## 3 Results

Out of the 71 patients included between 2019 and 2022, 54 had MCI and 17 had SCD; 60 answered the MBI‐Checklist (13 SCD and 47 MCI patients). General characteristics are described in Table 1. Age, education level, BDI scores and the values of A42 and T‐tau had normal distribution. P‐tau, BAI scores and all domains of MBI‐C had non‐parametric distribution. There were no significant differences between the groups considering the analysed variables. The results of variance analysis are also highlighted in Table 1.

**TABLE 1:** Sociodemographic variables and variance analysis.

	Groups		*p*
	SCD	MCI	
	Mean ± SD	Mean ± SD	
Age	63 ± 7.0	66 ± 6.0	0.062
Education (years)	14 ± 4.0	13 ± 5.0	0.417
Sex (female, %)	64.7%	63%	0.896
BAI	5 ± 3	6 ± 7	0.910
BDI	10 ± 10	9 ± 6	0.737
Total MBI‐C	9 ± 7	9 ± 8	0.906
MBI‐C (A)	3 ± 3	2 ± 3	0.681
MBI‐C (B)	4 ± 4	3 ± 3	0.736
MBI‐C (C)	2 ± 2	3 ± 3	0.279
MBI‐C (D)	0 ± 1	1 ± 1	0.190
MBI‐C (E)	0 ± 0	0 ± 1	0.096
A42	1056.15 ± 313.53	979.47 ± 425.26	0.531
T‐tau	190.31 ± 62.06	216.99 ± 74.31	0.185
P‐tau	15.72 ± 6.85	18.70 ± 7.63	0.133

Abbreviations: (A) to (E), MBI‐C domains; A42, amyloid‐beta 42 peptide; BAI, beck anxiety inventory; BDI, beck depression inventory; MBI‐C, mild behavioural impairment checklist; MCI, mild cognitive impairment; P‐tau, hyperphosphorylated tau; SCD, subjective cognitive decline; SD, standard deviation; T‐tau, total tau.

A significant correlation (*p*: 0.006) was observed between MBI‐C C domain and the concentrations of CSF amyloid‐beta peptide (Table 2).

**TABLE 2:** Spearman's correlation between AD fluid biomarkers and neuropsychiatric symptoms adjusted for sex and age.

	BDI‐II	BAI‐II	Total score MBI‐C	A domain	B domain	C domain	D domain	E domain
A42	0.033	0.056	−0.177	−0.37	−0.056	−0.366*	−0.138	−0.12
T‐tau	−0.221	−0.105	−0.112	−0.77	−0.032	−0.215	−0.136	0.152
P‐tau	−0.195	−0.159	−0.055	−0.058	−0.06	−0.068	−0.072	0.104

Abbreviations: A42, amyloid‐beta 42; AD, Alzheimer's disease; A–E domains, subitems of MBI‐C; BAI, Beck Anxiety Inventory; BDI, Beck Depression Inventory; MBI‐C, mild behavioural impairment—checklist; P‐tau, hyperphosphorylated tau; T‐tau, total tau.

* *p <* 0.01. Classification of the Spearman correlation coefficient: 0.6 < *ρ* < 1.0 = strong; 0.4 < *ρ* < 0.6 = moderate; 0.2 < *ρ* < 0.4 = weak.

## 4 Discussion

The results of the analysis in this study showed negative correlations between domain C of the MBI‐C and CSF A42 levels. This result corroborates the relation of the symptoms assessed by this domain and AD pathophysiology, considering that in AD, this biomarker is also decreased in CSF. There was no correlation between any MBI domain and P‐tau ou T‐tau, and it may be due to low tau levels in this population. This sample may represent individuals at very early AD stages along the AD continuum.

To understand this result and the literature findings regarding this thematic, it is important to record that each MBI‐C domain evaluates a set of NPSs. The A domain assesses decreased motivation symptoms, like apathy and indifference. In turn, the B domain evaluates affective symptoms, such as anxiety, irritability and dysphoria, etc. The MBI‐C C domain assesses impulse dyscontrol symptoms, such as agitation, impulsivity, aggression, recklessness and abnormalities in reinforcement and reward systems. The D domain rates social inappropriateness symptoms, like lack of empathy, rigidity, loss of social tact and exaggeration of previous personality traits. The MBI‐C E domain measures symptoms of abnormalities in perception or thought content just like hallucinations, delusions and suspiciousness [9]. There is no consensus about a cut‐off score; however some studies suggest that a total score of 6.5 points for MCI, 8.5 for SCD and 6 for cognitive unimpaired (CU) people has good sensitivity and specificity. In addition, no study has indicated a cut‐off score for a specific domain [8].

PAN et al. (2021) verified the prevalence of MBI domains in SCD and MCI patients in their pioneer systematic review [23]. The included studies did not apply only MBI‐C for the measurement of MBI domains, but also used the NPI‐Q, a scale elaborated to assess NPS in individuals with dementia [11] and ISTAART criteria for MBI [7], which differ from the present work analyses. Despite the considerable variability of the studies in the research, this review showed that impulse dyscontrol symptoms were present in 31.42% of SCD subjects and 29.72% of MCI subjects. A, B, D and E domains were also present in SCD (11.69%, 32.03%, 8.91% and 2.42%, respectively) and MCI (29.23%, 41.16%, 15.78% and 7.53% respectively) subjects. B and C domains were the most prevalent in both groups.

In a recent review, when it comes to using NPI and NPI‐Q to measure NPS, there are discrepant findings [24]. For instance, Sun et al. [36] identified an association between MBI total score and PET amyloid burden in CN and MCI individuals; however Chan et al. [41] did not find any association between PET amyloid or white matter hyperintensities (WMH) and NPS in CU individuals [25, 26]. Miao et al. [42] described that in a group of CU and MCI individuals, a higher MBI score was related to lower Ab42/Ab40 and affective dysregulation, but neither impulse dyscontrol nor impaired drive/motivation was related to Ab42/Ab40 [27].

According to Ruthirakuhan et al. [43], MBI predicted the progression to both the clinical and the neuropathologically confirmed diagnosis of AD; psychosis was the MBI domain with the largest effect on this association, followed by social inappropriateness, impulse dyscontrol, decreased motivation and affective dysregulation [28]. Another study by Gonzalez‐Bautista et al. [44] identified that the MBI domains of abnormal perception and social inappropriateness are linked to steeper increases of plasma P‐tau_181_ in community‐dwelling individuals, even though affective dysregulation and impulse dyscontrol were much more prevalent [29].

Studies using MBI‐C to analyse the prevalence of each MBI domain in SCD and MCI individuals and its relation to AD biomarkers are scarce [30, 31, 32]. Unlike our result, Johansson et al. [17] found significant correlations between MBI‐C C and B domains and CSF P‐Tau_181_ in amyloid beta positive CU individuals [30]. However, Lussier et al. [22] verified a positive association between MBI‐C total scores and PET amyloid but not tau in cognitively normal (CN) subjects; MBI‐C B and C domains were the most prevalent [32]. Another study by Cassidy et al. [45], found that in tau‐positive participants, higher locus coeruleus signal predicted NPS severity independently of tau burden, amyloid‐beta burden, and cortical grey matter volume. Impulse dyscontrol (MBI‐C C domain) was most correlated with this finding. Considering this context, the present study is a pioneer in this kind of investigation, and further studies are required to confirm our findings. Thus, the impression is that in preclinical AD, MBI domains such as affective dysregulation and impulse dyscontrol are more prevalent, but abnormal perception and social inappropriateness seem more linked to the progression of the disease.

In addition to MBI‐C, the present work also analysed the correlation of BAI and BDI with AD CSF biomarkers. Besides the lack of significant results in our study, there is evidence in the literature that anxiety and depression are present in the AD continuum. Ismail et al. [12] demonstrated in their meta‐analysis that depression was present in 32% of MCI patients. The included studies applied variable tools to determine depression diagnosis, but the method did not interfere significantly in the analysis results [33].

When it comes to SCD, depression is prevalent in this population. In Liew [21] prospective study, 22.18% of SCD participants were also depressed. The same study evidenced that the association of SCD with depression further increased the cognitive decline in comparison to SCD or depression separately [34]. However, it is still uncertain whether depression and SCD are parts of different pathologic processes or whether SCD is one of the manifestations of a depressive syndrome [35]. The clarification of the association of AD biomarkers with depressive symptoms in this group could be useful to explain the biological basis of this relation.

A similar context is applicable to anxiety symptoms in subjects with SCD. Anxiety is also prevalent in the SCD population (16.88%), increases the risks of cognitive decline and there is not a complete comprehension of the limits between pathophysiological processes inherent to anxiety and SCD [36].

When it comes to MCI, the reported prevalence of anxiety varied between 9.9% and 52% [37]. Smith et al. [35] conducted a study using data from the WHO Study on global AGEing and adult health (SAGE) to examine the association between anxiety and MCI in individuals aged 50 years and older. They analysed data from 32 715 individuals from multiple countries and found a positive association between anxiety and MCI across all countries studied [38].

Ramakers et al. [31] studied MCI individuals and verified an association between anxiety symptoms and CSF abnormal levels of amyloid beta and T‐tau. However, they did not find any correlation between the biomarkers and depressive symptoms [39]. A meta‐analysis also did not find significant correlations between depression and depressive symptoms and CSF biomarkers considering MCI subjects [40]. There is no current research studying these associations in SCD subjects specifically.

This study has some strengths and weaknesses. There are no studies evaluating the correlation between AD CSF biomarkers and the Brazilian version of MBI‐C, ensuring the relevance of this research to advance the studies on this topic in Brazil. Additionally, this research investigates NPSs and their relation to AD CSF biomarkers in patients with very early AD, a topic that remains scarce in the current literature.

The present work used MBI‐C self‐responded scores, configuring a probable bias of the method, considering that this checklist was originally made to be answered by patients' caregivers. In addition, the small sample of SCD subjects may have interfered in the sensitivity of the results.

The study identified a significant correlation between domain C of the MBI‐C and CSF beta‐amyloid peptide analysing Brazilian patients with SCD and MCI. The results reinforce the hypothesis of the relationship between impulsivity symptoms and amyloid pathology. Further studies involving the correlations between MBI and NPSs with AD CSF biomarkers are necessary.

## Funding

This work was supported by Fundação de Amparo à Pesquisa do Estado de São Paulo (FAPESP) (18/15571‐7).

## Conflicts of Interest

The authors declare no conflicts of interest.

## Data Availability

The data that support the findings of this study are available on request from the corresponding author. The data are not publicly available due to privacy or ethical restrictions.
